# Primary and secondary data in emergency medicine health services research – a comparative analysis in a regional research network on multimorbid patients

**DOI:** 10.1186/s12874-023-01855-2

**Published:** 2023-02-04

**Authors:** Anna Schneider, Andreas Wagenknecht, Hanna Sydow, Dorothee Riedlinger, Felix Holzinger, Andrea Figura, Johannes Deutschbein, Thomas Reinhold, Mareen Pigorsch, Ulrike Stasun, Liane Schenk, Martin Möckel

**Affiliations:** 1 Charité – Universitätsmedizin Berlin, corporate member of Freie Universität Berlin and Humboldt-Universität zu Berlin, Institute of Medical Sociology and Rehabilitation Science, Berlin, Germany; 2 Charité – Universitätsmedizin Berlin, corporate member of Freie Universität Berlin and Humboldt-Universität zu Berlin, Division of Emergency Medicine, Campus Virchow-Klinikum and Campus Charité Mitte, Berlin, Germany; 3 Charité – Universitätsmedizin Berlin, corporate member of Freie Universität Berlin and Humboldt-Universität zu Berlin, Institute of Social Medicine, Epidemiology and Health Economics, Berlin, Germany; 4 Charité – Universitätsmedizin Berlin, corporate member of Freie Universität Berlin and Humboldt-Universität zu Berlin, Institute of General Practice, Berlin, Germany; 5 Charité – Universitätsmedizin Berlin, corporate member of Freie Universität Berlin and Humboldt-Universität zu Berlin, Department of Psychosomatic Medicine, Berlin, Germany; 6 Charité – Universitätsmedizin Berlin, corporate member of Freie Universität Berlin and Humboldt-Universität zu Berlin, Institute of Biometry and Clinical Epidemiology, Berlin, Germany

**Keywords:** Health services research, Primary and secondary data, Emergency medicine

## Abstract

**Background:**

This analysis addresses the characteristics of two emergency department (ED) patient populations defined by three model diseases (hip fractures, respiratory, and cardiac symptoms) making use of survey (primary) and routine (secondary) data from hospital information systems (HIS). Our aims were to identify potential systematic inconsistencies between both data samples and implications of their use for future ED-based health services research.

**Methods:**

The research network EMANET prospectively collected primary data (*n*=1442) from 2017-2019 and routine data from 2016 (*n*=9329) of eight EDs in a major German city. Patient populations were characterized using socio-structural (age, gender) and health- and care-related variables (triage, transport to ED, case and discharge type, multi-morbidity). Statistical comparisons between descriptive results of primary and secondary data samples for each variable were conducted using binomial test, chi-square goodness-of-fit test, or one-sample t-test according to scale level.

**Results:**

Differences in distributions of patient characteristics were found in nearly all variables in all three disease populations, especially with regard to transport to ED, discharge type and prevalence of multi-morbidity. Recruitment conditions (e.g., patient non-response), project-specific inclusion criteria (e.g., age and case type restrictions) as well as documentation routines and practices of data production (e.g., coding of diagnoses) affected the composition of primary patient samples. Time restrictions of recruitment procedures did not generate meaningful differences regarding the distribution of characteristics in primary and secondary data samples.

**Conclusions:**

Primary and secondary data types maintain their advantages and shortcomings in the context of emergency medicine health services research. However, differences in the distribution of selected variables are rather small. The identification and classification of these effects for data interpretation as well as the establishment of monitoring systems in the data collection process are pivotal.

**Trial registration:**

DRKS00011930 (EMACROSS), DRKS00014273 (EMAAGE), NCT03188861 (EMASPOT)

**Supplementary Information:**

The online version contains supplementary material available at 10.1186/s12874-023-01855-2.

## Background

Emergency departments (EDs) are challenging research environments. Acutely and life-threateningly ill patients, high patient traffic, relatively short patients’ length of stay, 24-hours operation time and opening hours, as well as symptom-based emergency care, require specific adaptations to the patient recruitment process for health services research projects [1, 2]. Research based on primary data (i.e., data that is collected for a research-specific purpose) is cost-intensive and prone to biases during data collection that might impair results’ generalizability, but is inevitable for certain research questions relying on valid real-life data from health care settings [3, 4]. Secondary data (i.e., data that is produced by third parties for their specific purpose) from hospital information systems (HIS) provide an easily accessible data source that includes an entire ED population [5]. However, these data are collected primarily for medical documentation and reimbursement and not for research purposes. Additionally, there is no uniform documentation standard or a standardized set of variables across the HIS data of different hospitals, at least in Germany [6].

The use of primary and secondary data is often discussed in terms of advantages and disadvantages of both types [5, 7–10]. Primary data bear the risk of bias, face validity problems regarding participants’ responses as well as the possibility of non-response. For longitudinal studies, Roos et al. argue that poor representativeness is due to the complexity of the participant recruitment process, the circumstances of the ‘initial contact’ often leading to non-participation and the difficulty of maintaining contact for follow-up interviews [11]. Patient consent for studies linking primary and secondary data is challenging and has effects on the representativeness of studies [12, 13]. Other studies using primary data demonstrate the effort and complexity of the recruitment process and describe strategies for achieving representative samples [14–17]. Secondary data, on the other hand, have a lower risk of bias and often reflect a high number of cases. Direct contact with participants for data collection is not necessary and non-response as well as loss to follow-up are not as prevalent as in primary data collection [11]. However, secondary data bear the risk of possible deficiencies in data validity and quality as those depend on complex coding processes and documentation discipline within the institution producing the data.

Whereas advantages and disadvantages of both data types for research are well known and have been critically discussed, comparative analysis of corresponding primary and secondary data in the ED setting are rare. A review of studies on drug effects using primary and secondary data by Prada-Ramallal et al. showed that differences between data types were almost never addressed as a (possible) cause for heterogeneous study results [18]. However, a few studies showed differences in frequency distributions of specific outcomes when comparing primary and secondary data types, e.g., concerning diagnoses and associated comorbidities [19] and cost estimates for primary care utilization [20].

### Goals of this investigation

For this analysis, we compared three primary data study populations with respective secondary data HIS populations regarding socio-structural (age, gender) and health- and care-related characteristics (triage category, transportation to ED, case and discharge type, multi-morbidity). Our research question was: What are potential implications of using primary and secondary data for analyzing care within EDs? In addition, our analysis aims to show potential insights from the comparison of both data types and methodological-practical suggestions derived from this investigation’s experiences.

## Methods

### Study design and setting

In this analysis, we investigated data from the regional research network EMANET (Emergency and Acute Medicine Network for Health Care Research Berlin). The overall scientific goal of EMANET is the identification, development, and implementation of measures for optimized health care of multi-morbid patients in emergency and acute care [21]. In the first funding period from 2016 to 2020, three mixed-methods studies were conducted in all of the eight interdisciplinary EDs in Berlin’s central administrative district Mitte. The participating hospitals differed with regard to ownership (state and federal authorities, denominational organizations, non-profit organizations), total numbers of hospital beds (from approximately 150 to 1,700), and yearly ED patient numbers (from 10,000 to 110,000). However, since all EDs of the respective city district were included in the EMANET network, the study ensured access to a group of ED patients representative for this specific urban area. The network’s aim was to investigate the specific characteristics of and healthcare provision for acute patients with one of three model diseases: (a) EMAAGE aimed at the emergency and follow-up health care of patients with hip fractures; (b) EMACROSS focused on cross-sectoral care provision for patients with respiratory diseases (with an initial focus on outpatients, which was expanded throughout the recruitment process to also include inpatients) [21, 22] and (c) EMASPOT targeted comorbid mental health conditions, such as depression and anxiety, in elderly patients with cardiac symptoms and diseases [23].

### Selection of participants

Inclusion into the primary data sample and extraction of secondary data was performed for patients in participating study EDs with at least one of the respective leading symptoms and diagnoses (see Additional Table 2) [24]. Model diseases and respective diagnoses were initially chosen from a publication on ambulatory care sensitive conditions (ACSC) in the German healthcare setting and adapted to the need of patient recruitment in the ED so that symptom diagnose codes were included [25]. ACSC are a group of common chronic and acute illnesses considered not to require inpatient treatment if appropriate ambulatory care is received [26]. The basis of our analysis consisted of three data types: (a) primary data from the three sub-studies with baseline surveys, as well as data from an electronic case report form (eCRF) for the period from 2017 to 2019, (b) a screening log that monitored and documented the recruitment process and reasons for non-participation for the period of the primary data collection, and (c) secondary data from the HIS of participating EDs for the year 2016. The HIS dataset included all ED visits of patients with respective study-related diagnoses and posed a complete representation of the relevant ED population in the specified time frame of one year.

### Data acquisition

For primary data collection, trained study personnel recruited patients between June 1, 2017 and June 28, 2019 during fixed time periods, generally on weekdays between 8 am and 5 pm, in all of the eight participating EDs. Occasionally, recruitment was extended to weekends or weekday evenings. Patients were interviewed by study nurses in the acute ED situation (EMASPOT, EMACROSS) or postoperatively on hospital wards (EMAAGE). Potential study participants were identified by study nurses via patient screening in participating EDs using data from the HIS. Inclusion criteria were project-specific leading symptoms and age (50+ years for EMASPOT and 18+ years for EMAAGE and EMACROSS). If necessary, medical, nursing, or administrative ED staff was consulted for the clarification of inclusion- and interview-relevant questions (e.g., regarding suspected diagnoses and leading symptoms or patients’ ability to be interviewed). Since the inclusion of patients in the primary data sample was based on leading symptoms and not on final and confirmed diagnoses, there was a possibility that a patient no longer possessed one of the study-relevant diagnoses at the end of his or her ED treatment. In these cases, respective patients were subsequently excluded from the study population. The screening process was documented in printed structured questionnaires, i.e., screening logs [4]. After inclusion and informed consent, study nurses interviewed patients for approximately 30 to 60 minutes with handheld tablets containing electronic versions of the study-specific questionnaires. Printed study materials were additionally available in German, English, Arabic, and Turkish language. All participants gave written permission for review of their individual electronic health records for study-specific aims.

For secondary data collection, HIS data of the eight EDs were retrieved for all patients that were treated in one of these EDs during the year 2016 and met the inclusion criteria of at least one of the EMANET sub-studies with respect to age and coded diagnoses according to the International Classification of Diseases (ICD), 10^th^ Revision [27] (see Additional Table 2). Since data were anonymized before extraction, patients’ consent was waived. All EDs received a list of predefined variables for extraction including patients’ sociodemographic information, diagnosis ICD-codes of ED and inpatient treatment, and parameters of ED care.

### Data management

Primary data collection in patient interviews was tablet-based and data was automatically transferred after entry to a Research Electronic Data Capture (REDCap) tool hosted at Charité – Universitätsmedizin Berlin. Each study participant received a unique pseudonym. Screening log data and administrative participant data was saved separately in a Microsoft Access database. Further information from patients’ electronic hospital files was manually entered by study nurses into a study-specific eCRF in another REDCap database using the respective participant pseudonym. The central data management of EMANET collated data sets by using participant pseudonyms.

Anonymized secondary data were prepared and delivered to the central data management of the coordinating unit of EMANET by the participating EDs, the information technology (IT) departments of the respective hospitals or the vendors of the respective HIS in CSV (comma-separated values) or Microsoft Excel files. Data delivery followed established data protection procedures on password-protected devices as described in the project’s data protection concept. The central data management checked all data for completeness and plausibility and linked all files of the participating EDs to yield one final data set with secondary data from all EDs. Due to varying documentation standards between the participating EDs, it was necessary to harmonize the data to establish comparability. HIS data were adapted according to data harmonization rules consented by a working group of EMANET researchers. These rules basically followed the data harmonization recommendations of the INDEED project [28]. All primary and secondary data was stored on servers of the Charité – Universitätsmedizin Berlin.

**Fig. 1:**
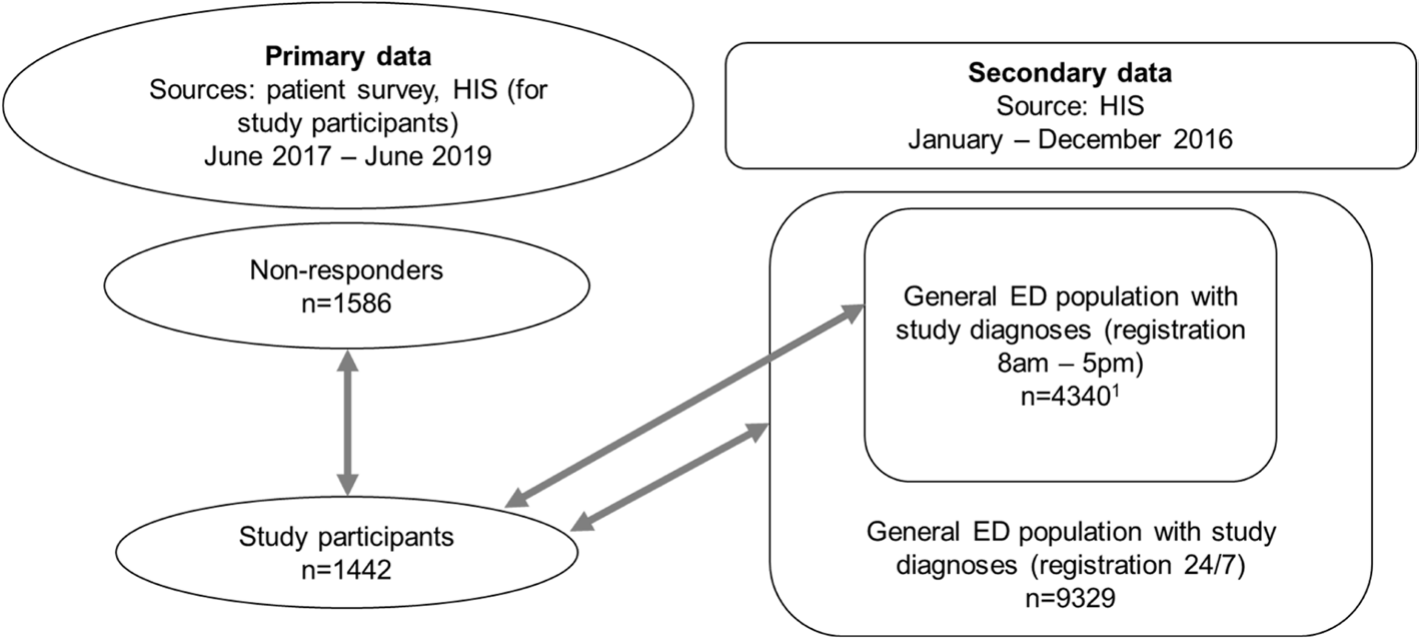
Illustration of primary and secondary data samples across the three study populations in EMANET. Legend: Ellipses depict the primary data sample and rectangles secondary data samples used for analyses. Arrows between shapes illustrate data samples which were compared numerically in this study. Numbers summarize patients with relevant study diagnoses for all three research projects (EMAAGE, EMACROSS, EMASPOT). ^1^The sample of the general ED population registered between 8am and 5pm excludes ED patients with study-related diagnoses from EMAAGE since patients were recruited into this study on wards following ED treatment without time restrictions. Abbreviations: ED: emergency department, HIS: hospital information system

For comparative analyses between primary and secondary data it was necessary to create two differently tailored datasets of secondary data. One secondary data set was adapted to the recruitment conditions and inclusion criteria of the primary data sample (see Fig. 1 and Additional Figs. 1, 2, 3 for each sub-study), i.e., secondary data for EMACROSS included only patients registered in EDs between 8 am and 5 pm analogous to patient recruitment times of the primary data sample and secondary data in EMASPOT only included patients with an age of 50 years or older and registered in EDs between 8 am and 5 pm. The second data set included all patients presenting with relevant diagnoses (for EMASPOT: age ≥ 50 years) independently from the time of ED admission.

### Measurements

In this analysis, the following variables were selected as suitable for comparison between data types: age, gender, triage category, transportation type, case type, discharge type, and multi-morbidity. The choice of variables is justified by their general availability within all eight HIS in the participating hospitals, the comparatively small number of missing data within variables, and their relevance to clinical routine in EDs. Except for multi-morbidity, all variables were generated by directly interviewing patients or by data extraction from each ED’s documentation system. Triage categories were structured according to the Manchester Triage System (MTS). Transportation to the ED was coded in walk-in (including patients accompanied by relatives), by ambulance services (transportation of non-urgent and mobility-impaired patients), by emergency medical services (EMS), and by EMS accompanied by an emergency physician. Case type was coded into in- or outpatient. Discharge type for inpatients was differentiated in discharge to home or an existing care arrangement, transfer to another hospital or other health care facility, death, or other. Multi-morbidity was defined according to van den Bussche et al. as three or more chronic diseases of a predefined list of ICD-codes [29]. For our comparative analyses, ICD-10 diagnoses documented in HIS and diagnoses reported by participants were used to determine multi-morbidity.

### Statistical analysis

The aim of the statistical analysis was to compare primary data and secondary data regarding their similarity in the distribution of various ED-related parameters. For descriptive statistics, the continuous variable (age) was characterized by its mean and standard deviation (SD). Categorical variables (gender, triage category, type of transportation to the ED, multi-morbidity, discharge type, case type) were summarized as numbers (percentages) of subjects. We computed 95% Wald confidence intervals for the distribution of percentage estimates of categorical variables for population proportions of n≥5. In order to estimate the distribution of variables in primary data in comparison to the ED population with respective diagnoses in secondary data, we calculated binomial tests, chi-square goodness-of-fit tests, or one-sample t-tests according to scale level. Available case analysis (pairwise deletion) in SPSS version 27 (IBM Inc.) was used for all analyses. In order to discuss differences between variable distributions in the study-specific primary and secondary data samples, primarily differences in point estimates and confidence intervals of each variable were consulted. Graphical distributions in the form of bar charts for multi-categorical variables (MTS category, transportation to ED, discharge type) are available in Additional Figs. 4, 5 and 6. We included all relevant items of the Strengthening the Reporting of Observational studies in Epidemiology (STROBE) Statement in this manuscript [30].

## Results

### Patients with hip fractures (EMAAGE): primary versus secondary data samples

In the population consisting of patients with hip fractures (EMAAGE; *n*=326 in primary data sample; *n*=439 in secondary data sample), the distributions of age and gender were similar between both samples (see Table 1). Although statistically significant, only minor differences were found in the triage category, i.e., slightly more patients with triage category 2 (“very urgent treatment”) were included in the primary cohort compared to the secondary data population (16.4% vs. 12.6%; see Additional Fig. 4). Concerning the other triage categories, we observed only small deviations between 1 percentage point (pp) and 2pp, rendering the distribution of patients in both populations between triage categories similar. Concerning case type, while all patients in the primary sample were hospitalized after ED treatment, 3.2% of patients from the secondary sample were coded as released after their ED stay in HIS data. The variables transport type, discharge type and multi-morbidity showed more profound differences. In transport categories, differences between 3pp and 5pp were found (see Additional Fig. 5). However, the proportion of patients transported by EMS was similar, i.e., the confidence interval included the estimate of the secondary data sample with respective diagnoses. The discharge type altered between cohorts, e.g., 16pp more patients in the primary data sample were transferred to another health care facility, while 13pp less patients were discharged home or to a pre-existing care arrangement (see Additional Fig. 6). Finally, the primary data sample included 8pp more multi-morbid patients compared to HIS data (65.3% vs. 57.4%).

**Table 1:** Description and statistical comparison of patient characteristics in primary and secondary data samples (EMAAGE)

Variable	Primary data sample (*n*=326) n (%, [95% CI])	Secondary data sample (*n*=439) n (%)	*p* value
Gender			.380
Male	107 (32.8, [27.7; 37.9])	140 (31.9)	
Female	219 (67.2, [62.1; 72.3])	299 (68.1)	
Age in years (mean (SD))	75.8 (12.1, [74.5; 77.1])	76.8 (13.6)	.132
Multi-morbidity: Yes	213 (65.3, [60.1; 70.5])	252 (57.4)	.002
MTS category^a^			.043
1 (immediate treatment)	1 (0.4)	6 (2.4)	
2 (very urgent treatment)	44 (16.4, [12.0; 20.8])	32 (12.6)	
3 (urgent treatment)	187 (69.8, [64.3; 75.3])	181 (71.5)	
4 (normal)	32 (11.9, [8.0; 15.8])	34 (13.4)	
5 (not urgent)	4 (1.5)	0 (0)	
Transportation to ED			<.001
Walk-in	9 (2.9, [1.0; 4.8])	26 (7.1)	
Non-urgent medically accompanied transport	49 (15.9, [11.8; 20.0])	76 (20.7)	
Emergency medical services	211 (68.3, [63.1; 73.5])	239 (65.1)	
EMS with emergency physician	40 (12.9, [9.2; 16.6])	26 (7.1)	
Case type			<.001
Outpatient	0 (0)	14 (3.2)	
Inpatient	326 (100)	425 (96.8)	
Discharge type			<.001
Home or existing care arrangement	100 (31.6, [26.5; 36.7])	191 (44.9)	
Transfer (to another hospital or health care facility)	206 (65.2, [59.9; 70.5])	206 (48.5)	
Deceased	8 (2.5, [0.8; 4.2])	24 (5.6)	
Other	2 (0.6)	4 (0.9)	

*Note*: 95% Wald confidence intervals were computed for population proportions of *n*≥5. *CI* Confidence interval, *ED* Emergency department, *EMS* Emergency medical services, *MTS* Manchester Triage System, *SD* Standard deviation; ^a^The chi-square goodness-of-fit test was conducted with an adjusted variable for MTS category containing categories 1 to 4 due to missing values in the secondary data sample

### Patients with respiratory diseases (EMACROSS): primary versus secondary data samples

In patients with respiratory diseases (EMACROSS; *n*=472 in primary data sample; *n*=1,563 in secondary data sample (presentation between 8am and 5pm), *n*=3,410 in secondary data sample (without time restriction)), characteristics between primary and secondary data samples showed pronounced differences in the comparison of deviations regarding pp and confidence intervals (see Table 2). However, the distribution of gender and multi-morbidity did not show any relevant differences in the three samples. Study participants (53.6 years) were on average six years younger than patients in the secondary data samples (59.9 years and 59.1 years, respectively). More patients were discharged home or to existing care arrangements (87.0%) than in the secondary data samples (80.3% and 79.7%, respectively; see Additional Fig. 6). Mortality was lower in the primary data sample (0.2%) than in the secondary data samples (8.3% and 7.7%, respectively). Accordingly, less patients in the triage categories 1-3, and correspondingly more patients in categories 4 and 5 were recruited (see Additional Fig. 4). The type of transport to the ED differed: primary data contained fewer patients who were transported by ambulance and EMS, but slightly more patients who were accompanied by an emergency physician, and far more walk-in patients (see Additional Fig. 5). Finally, EMACROSS recruited substantially more outpatient cases compared to the proportion of outpatients in the secondary data samples.

**Table 2:** Description and statistical comparison of patient characteristics in primary and secondary data samples (EMACROSS)

Variable	Primary data sample (*n*=472) n (%, [95% CI])	Secondary data sample (presentation between 8am and 5pm) (*n*=1563) n (%)	*p* value	Secondary data sample (presentation throughout the day) (*n*=3410) n (%)	*p* value
Gender			.453		.298
Male	251 (53.2, [48.7; 57.7])	826 (52.8)		1860 (54.5)	
Female	221 (46.8, [42.3; 51.3])	737 (47.2)		1550 (45.5)	
Age in years (mean (SD))	53.6 (19.1, [51.9; 55.3])	59.9 (19.3)	<.001	59.1 (19.7)	<.001
Multi-morbidity: Yes	186 (39.4, [35.0; 43.8])	626 (40.1)	.398	1375 (40.3)	.365
MTS category			<.001		<.001
1 (immediate treatment)	4 (0.9)	36 (4.6)		90 (4.9)	
2 (very urgent treatment)	106 (23.2, [19.3; 27.1])	219 (28.2)		502 (27.3)	
3 (urgent treatment)	155 (34.0, [29.7; 38.3])	303 (39.0)		717 (39.0)	
4 (normal)	173 (37.9, [33.4; 42.4])	207 (26.7)		500 (27.2)	
5 (not urgent)	18 (3.9, [2.1; 5.7])	11 (1.4)		29 (1.6)	
Transportation to ED			<.001		<.001
Walk-in	291 (63.0, [57.6; 66.4])	445 (42.0)		1043 (42.9)	
Non-urgent medically accompanied transport	29 (6.4, [4.2; 8.6])	219 (20.7)		422 (17.3)	
Emergency medical services	111 (24.7, [20.8; 28.6])	355 (33.5)		834 (34.3)	
EMS with emergency physician	31 (6.9, [4.6; 9.2])	41 (3.9)		134 (5.5)	
Case type			<.001		<.001
Outpatient	289 (61.2, [56.8; 65.6])	613 (41.7)		1315 (40.7)	
Inpatient	183 (38.8, [34.4; 43.2])	857 (58.3)		1917 (59.3)	
Discharge type			<.001		<.001
Home or existing care arrangement	387 (87.0, [83.9; 90.1])	756 (80.3)		1714 (79.7)	
Transfer (to another hospital or health care facility)	41 (9.2, [6.5; 11.9])	103 (10.9)		258 (12.0)	
Deceased	1 (0.2)	78 (8.3)		165 (7.7)	
Other	16 (3.6, [1.9; 5.3])	4 (0.4)		13 (0.6)	

*Note*: 95% Wald confidence intervals were computed for population proportions of *n*≥5. *CI* Confidence interval, *ED* Emergency department, *EMS* Emergency medical services, *MTS* Manchester Triage System, *SD* Standard deviation

### Patients with cardiac symptoms and diseases (EMASPOT): primary versus secondary data samples

In the third project EMASPOT (*n*=644 in primary data sample; *n*=2,777 in secondary data sample (presentation between 8am and 5pm), and *n*=5,480 in secondary data sample (without time restriction) patients with cardiac symptoms and diseases of comorbid mental health conditions (MHCs) were screened and recruited. Differences in percentage points in the distribution of patient characteristics in primary and secondary data samples were rather small, although statistically significant (see Table 3). Data types did not differ concerning case type. Study participants were slightly younger (68.4 years) than the secondary data samples (69.8 years and 69.5 years, respectively). Slightly less patients from triage category 1 were recruited into the primary sample (0.5%) in comparison to the secondary data sample (1.4% and 2.4%, respectively; see Additional Fig. 4). Small differences of 3pp to 4pp were found for discharge types; however, no specific direction or recognizable structure was detectable (see Additional Fig. 6). The EMASPOT sample showed a clear difference in gender distribution, where 7pp more men were included in the primary data sample. As in the other sub-studies, moderate differences between primary and secondary data were found in the distribution of transport type (see Additional Fig. 5). The largest discrepancy of 14pp between populations was found in the prevalence of multi-morbidity with higher frequency for study participants (68.3%) in comparison to secondary data samples (54.1% and 52.6%, respectively).

**Table 3:** Description and statistical comparison of patient characteristics in primary and secondary data samples (EMASPOT)

Variable	Primary data sample (*n*=644) n (%, [95% CI])	Secondary data sample (presentation between 8am and 5pm) (*n*=2777) n (%)	*p* value	Secondary data sample (presentation throughout the day) (*n*=5480) n (%)	*p* value
Gender			<.001		<.001
Male	376 (58.4, [54.6; 62.2])	1434 (51.6)		2774 (50.6)	
Female	268 (41.6, [37.8; 45.4])	1343 (48.4)		2706 (49.4)	
Age in years (mean (SD))	68.4 (10.8, [67.6; 69.2])	69.8 (11.1)	.001	69.5 (11.1)	.008
Multi-morbidity: Yes	440 (68.3, [64.7; 71.9])	1501 (54.1)	<.001	2881 (52.6)	<.001
MTS category			.027		.003
1 (immediate treatment)	3 (0.5)	22 (1.4)		77 (2.4)	
2 (very urgent treatment)	225 (36.8, [33.0; 40.6])	579 (35.9)		1121 (35.0)	
3 (urgent treatment)	279 (45.7, [41.8; 49.6])	721 (44.8)		1422 (44.5)	
4 (normal)	93 (15.2, [12.4; 18.0])	275 (17.1)		549 (17.2)	
5 (not urgent)	11 (1.8, [0.7; 2.9])	14 (0.9)		30 (0.9)	
Transportation to ED			<.001		<.001
Walk-in	320 (52.8, [48.8; 56.8])	1106 (49.9)		2072 (46.5)	
Non-urgent medically accompanied transport	44 (7.3, [5.2; 9.4])	275 (12.4)		458 (10.3)	
Emergency medical services	170 (28.1, [24.5; 31.7])	672 (30.3)		1506 (33.8)	
EMS with emergency physician	72 (11.9, [9.3; 14.5])	162 (7.3)		422 (9.5)	
Case type			.432		.368
Outpatient	215 (33.4, [29.8; 37.0])	907 (33.0)		1860 (34.1)	
Inpatient	429 (66.6, [63.0; 70.2])	1840 (67.0)		3587 (65.9)	
Discharge type			<.001		<.001
Home or existing care arrangement	540 (88.2, [85.6; 90.8])	1643 (85.0)		3242 (84.6)	
Transfer (to another hospital or health care facility)	49 (8.0, [5.9; 10.1])	220 (11.4)		436 (11.4)	
Deceased	2 (0.3)	61 (3.2)		141 (3.7)	
Other	21 (3.4, [2.0; 4.8])	8 (0.4)		14 (0.4)	

*Note*: 95% Wald confidence intervals were computed for population proportions of *n*≥5. *CI* Confidence interval, *ED* Emergency department, *EMS* Emergency medical services, *MTS* Manchester Triage System, *SD* Standard deviation

### Non-responder analysis in the primary data sample and missing data

Of all eligible patients, 56.3% in EMAAGE, 45.7% in EMACROSS and 45.4% in EMASPOT gave consent to participate in the respective studies. Reasons for non-participation of eligible patients were described elsewhere as a summary for all three study populations [24]. Gender and age – the only available categories for comparison in non-responders and participants – showed slight differences. In EMAAGE, proportionally more women participated. In EMASPOT and EMACROSS, participants were younger than non-responders (see Additional Table 1).

Missing values in primary data samples and in the complete secondary data sample (with presentations throughout the day) applied to MTS category, transportation to ED, and discharge type (see Additional Table 3). Furthermore, data on case type was missing in EMACROSS and EMASPOT. Analyses of missing data in the secondary data sample revealed that information was partly not available (in the required format) from all eight ED HIS for the above listed variables: data on case type was not available in one ED; transportation to the ED and discharge type in two EDs; and MTS category in four EDs. Concerning the amount of missing values at the ED level, all of the above listed variables were supplied by three EDs, one missing variable each was observed in two ED HIS datasets, two missing variables each in a further two ED HIS datasets and three missing variables were observed in one ED HIS dataset.

## Discussion

This contribution’s novelty lies in the comparison of primary and secondary data in the emergency medicine health services research context and its inclusion of three different patient populations and respective indications from eight EDs, which is unique so far. Mostly minor, although statistically significant, differences in distributions of patient characteristics between primary and secondary data samples were found in most variables and for all three sub-studies. *Age* and *gender* distributions in study participants mostly reflected the secondary data sample which was also reported in similar trial studies [31].

Differences in patient and case characteristics can be attributed to recruitment conditions, study-specific inclusion criteria and the modus operandi of documentation in hospitals. One of the central reasons for the observed differences in data samples are the specifics of the recruitment situation and process of the three sub-studies, which has also been argued by Roos et al. in the context of longitudinal studies [11]. The effects of recruitment practices for the composition of study populations in health care research are discussed broadly. Some trial studies investigated barriers to patient recruitment, such as migration background, language barriers, cognitive characteristics that make informed consent difficult or the perceived lack of benefits for the patients [32–37]. The issue of recruitment barriers and their effects on study population composition are certainly important when studies operate with the goal of achieving certain case numbers and response rates. Identified further factors influencing recruitment were, e.g., certain communication channels (telephone vs. mail) [38], specific time points of recruitment [39], and the recruitment experience of study nurses [40].

Our analysis focused on patient characteristics and specific features of the recruitment situation. Generally, time restrictions in EMACROSS and EMASPOT did not generate meaningful differences with regard to the distribution of characteristics in primary and secondary data samples. However, in EMAAGE retrospective patient inclusion was practiced, so that in this sample the time of presentation to the ED was irrelevant. In cardiac patients, the prolonged stay on the Chest Pain Unit (CPU) of the ED may have helped to include patients during working hours that initially presented during night hours. Given the almost similar distributions of patient characteristics between samples, we conclude that restricting study recruitment to specific times of the day does not hamper the inclusion of a patient population similar to the target population. This finding appears to be a special feature and novelty of our contribution. Whether this feasibility of comparisons as well as the seemingly negligible impact of time restrictions to recruitment can be generalized to other clinical settings is beyond the scope of this article. As available literature suggests, recruiting and data collection heavily depend on the properties of certain settings [41–43].

The effect of the recruitment process is particularly noticeable for participants with respiratory diseases in EMACROSS whose characteristics differed more profoundly from the secondary data sample with respiratory diseases. Concerning the distribution of patients’ *triage categories*, severely ill and acute patients in category 1 were less often included in the primary study sample. Recruitment of patients for interviews of 30 to 60 minutes who are in need of immediate treatment is not feasible for medical and ethical reasons. Differences between populations found in triage categories therefore can be regarded as unavoidable. Concerning EMACROSS, recruitment might have been additionally hampered by patients’ physical inability to conduct an interview due to shortness of breath or respiratory therapy in the ED. We observed that older patients with respiratory complaints were more likely to be non-responders, which might have influenced the age distribution in the recruited population. As studies on hospice patients [31, 44, 45] or patients in stressful situations [46] argued in a similar way, primary data collection might be inappropriate or at least comes with a higher share of nonresponse, if patients suffer from certain illnesses. The same reasoning generally applies to studying diseases that affect patients’ communication skills. Thus, if the importance of particularly severely ill patients is relevant to the research question, recourse to HIS data may be more appropriate.

Inclusion criteria and respective changes during the recruitment process are of particular relevance for the total composition of a population. Participants in EMAAGE and EMASPOT reproduced the distribution of *ambulatory and inpatient* stays in the secondary data sample with respective diagnoses. The overrepresentation of ambulatory participants (and thus associated surplus of younger and healthier patients) in EMACROSS is explained by the project’s initial inclusion criteria focusing on outpatient ED patients. Participants of all sub-studies slightly differed with regard to *discharge types* from the secondary data samples. The difference in the number of deceased patients in EMACROSS and respiratory ED patients in general might be explained by the fact that this sub-study recruited mostly younger patients with ambulatory health care needs and rather average to low acuity measured by triage categories [47].

In EMAAGE, we observed an overrepresentation of patients who were transferred to other health care facilities in the primary data sample. This might be due to the focus of the study personnel on patients’ final care arrangements documented in electronic patient files while HIS data only captures the most immediate discharge type after hospital treatment, e.g., discharge home. This indicates the relevance of documentation routines and data production practices in patient surveys and routine data. This was even more obvious in the case of multi-morbidity, which is a generally complex variable [48]. Multi-morbidity was the variable with the most pronounced differences between the data sets in EMASPOT and EMAAGE with higher rates of multi-morbid patients in the primary data sample. This might be explained by two aspects: The primary data set was tailored to detect certain comorbidities that are not systematically documented in ED diagnoses. Especially for ambulatory ED patients in the secondary data sample, only diagnoses relevant for ED treatment are documented in the HIS. Thus, comorbid conditions might not have been systematically documented by healthcare personnel, as other studies also pointed out [29]. The definition of multi-morbidity applied in this data sample is dependent on thorough ICD-coding [29, 48, 49]. Thus, using ED diagnoses from HIS for determining patient multi-morbidity is potentially less suitable, since relevant diagnoses might be lacking and comorbidities are also often recorded in form of free text. Therefore, prevalence of multi-morbidity in ambulatory ED patients might be underestimated. In primary data collection for research purposes, study personnel cannot only inquire relevant diagnoses from patients themselves, but also search through the electronic patient file in HIS on past hospital stays, physician’s letters, and other sources of information. This argument is in line with reviews that have examined the construction of the variable multi-morbidity [50]. We complement the point made by Stirland et al. [50] by saying that the choice of data source is relevant and needs to be critically reflected when doing research on complex variables like multi-morbidity. However, the failure to diagnose and document specific conditions in the ED, e.g., mental health disorders, is another relevant point when considering the reliability of both primary and secondary data concerning completeness of diagnoses [23, 49].

Finally, with regard to patients’ *transport to the ED*, inconclusive differences were observed in all transport categories between primary and secondary data populations. No content-related explanation for these differences was found, thus indicating that recruitment in our study failed to reproduce the actual pattern of patients’ transport to the ED.

### Limitations

Our research combines comprehensive data of two types on three ED patient populations with common model diseases. Nevertheless, our analyses are subject to limitations. Firstly, samples of primary and secondary data were drawn from two different time periods due to research-practical reasons and availability of secondary data. This might have influenced absolute numbers in sample composition. However, no changes in the relative distribution of patient- and care-related characteristics in participating EDs should have occurred in the rather short period between 2016 and 2019, as there were no major changes in the prevalence of studied (mostly chronic) diseases, medical guidelines for ED treatment of these diseases, and in the structure or processes of ED care in Germany. Secondly, some variable categories in primary and secondary data sets originally differed and were thus harmonized retrospectively for comparative analyses, which might have introduced minor distortions of results. Thirdly, three variables (triage category, transportation to ED, and discharge type) showed high proportions of missing values, especially in secondary data, which might have influenced results. Reasons for high missing values in secondary data samples were mainly due to the fact that some EDs did not transmit data on certain variables, e.g., because this information was not collected in the respective ED at all (no mandatory field in documentation forms) or information was not collected systematically for every ED patient. If the amount of missing secondary data from specific study EDs would be systematically associated with the above-mentioned ED factors, the distribution of the respective variables in our analysis of secondary data might be biased. However, from descriptive analysis of ED features and the pattern of the amount of missing values per ED, no systematic bias in this direction became evident so that we can reasonably assume that missing values in our datasets occurred completely at random. Lower missing values in primary data in the same variables might point to the advantage of primary data collection by trained study nurses, who closely observed the care process of study participants during recruitment and manually retrieved not readily available information from all electronic documentation in the patient file. Fourthly, identification of cases in primary and secondary data was different (leading symptoms in primary recruitment and diagnoses in secondary data), which might have affected the comparability of populations. Lastly, the secondary data sample consists of cases from eight EDs. However, the number of patients treated in each ED differs vastly between EDs. As documentation of variables was not harmonized prior to data extraction, systematic differences may occur.

## Conclusions

Overall this articles shows, that the comparison of patient populations in primary and secondary data samples can provide insights into the advantages and shortcomings of both data types for health services research in emergency medicine. Complete secondary data thus allow to assess and to verify the composition of primary data samples if the same study inclusion criteria are applied to both samples and data sets are adjusted for comparison. Overall differences between primary and secondary data samples are evident in our patient populations but comparably small. Observed differences in patient characteristics of the primary data sample might have been influenced by recruitment practices (e.g., non-response, length and type of survey administration), project-specific inclusion criteria (e.g., language and cognitive requirements for study participation, focus on specific case types) and differing documentation rationales. Nevertheless, primary data allow a comprehensive and detailed collection of information on specific patient groups. The higher workload from patient recruitment and resulting lower sample sizes in primary datasets may be disadvantages. In contrast, the secondary data sample depicts the full population of ED patients with respective diagnoses in a specific time frame, although this data type bears the risk of incomplete information due to missing values or non-usable data formats in HIS documentation.

The aim of health services research studies is to depict real-life conditions of health care provision in certain patient groups or settings. Future research studies with primary data collection should thus additionally establish close concomitant monitoring practices during patient recruitment, in order to timely detect potential deviations from targeted sample characteristics. Additionally, our analysis has shown the need for systematic, harmonized and complete secondary data documentation in hospital information systems for health services research purposes.

## Supplementary Information

**Additional file 1: Figure 1.** Illustration of primary and secondary data samples of the study populations in EMAAGE.

**Additional file 2: Figure 2.** Illustration of primary and secondary data samples of the study populations in EMACROSS.

**Additional file 3: Figure 3.** Illustration of primary and secondary data samples of the study populations in EMASPOT.

**Additional file 4: Figure 4.** Graphical distribution of MTS categories in primary and secondary data samples in EMAAGE, EMACROSS, and EMASPOT.

**Additional file 5: Figure 5.** Graphical distribution of transportation to ED in primary and secondary data samples in EMAAGE, EMACROSS, and EMASPOT.

**Additional file 6: Figure 6.** Graphical distribution of discharge type in primary and secondary data samples in EMAAGE, EMACROSS, and EMASPOT.

**Additional file 7: Table 1.** Description of non-responders and study participants regarding gender and age from screening logs.

**Additional file 8: Table 2.** Inclusion criteria for the three EMANET sub-studies EMAAGE, EMACROSS, and EMASPOT.

**Additional file 9: Table 3.** Description of missing data in primary and secondary data samples in MTS category, transportation to ED, discharge type, and case type.

## Data Availability

Based on the data protection concept, positively evaluated by the Charité data protection office in 2017, the data set cannot be entirely published. Anonymized subsets of data are available from the corresponding authors on reasonable request.

## Abbreviations

ED: Emergency department
HIS: Hospital information system
ACSC: Ambulatory care sensitive conditions
eCRF: Electronic case report form
ICD: International Classification of Diseases
REDCap: Research Electronic Data Capture
MTS: Manchester Triage System
EMS: Emergency medical services
SD: Standard deviation
MHC: Mental health condition
CPU: Chest Pain Unit
