# Supplementary material for: Primary and secondary data in emergency medicine health services research – a comparative analysis in a regional research network on multimorbid patients
Source: BMC Med Res Methodol. 2023 Feb 4;23:34. doi: 10.1186/s12874-023-01855-2 (PMC9898937; doi:10.1186/s12874-023-01855-2)
Supplement: Supplementary file 1 — Additional file 1: Figure 1. Illustration of primary and secondary data samples of the study populations in EMAAGE. [file 12874_2023_1855_MOESM1_ESM.docx]

Additional Figure 1: Illustration of primary and secondary data samples of the study populations in EMAAGE


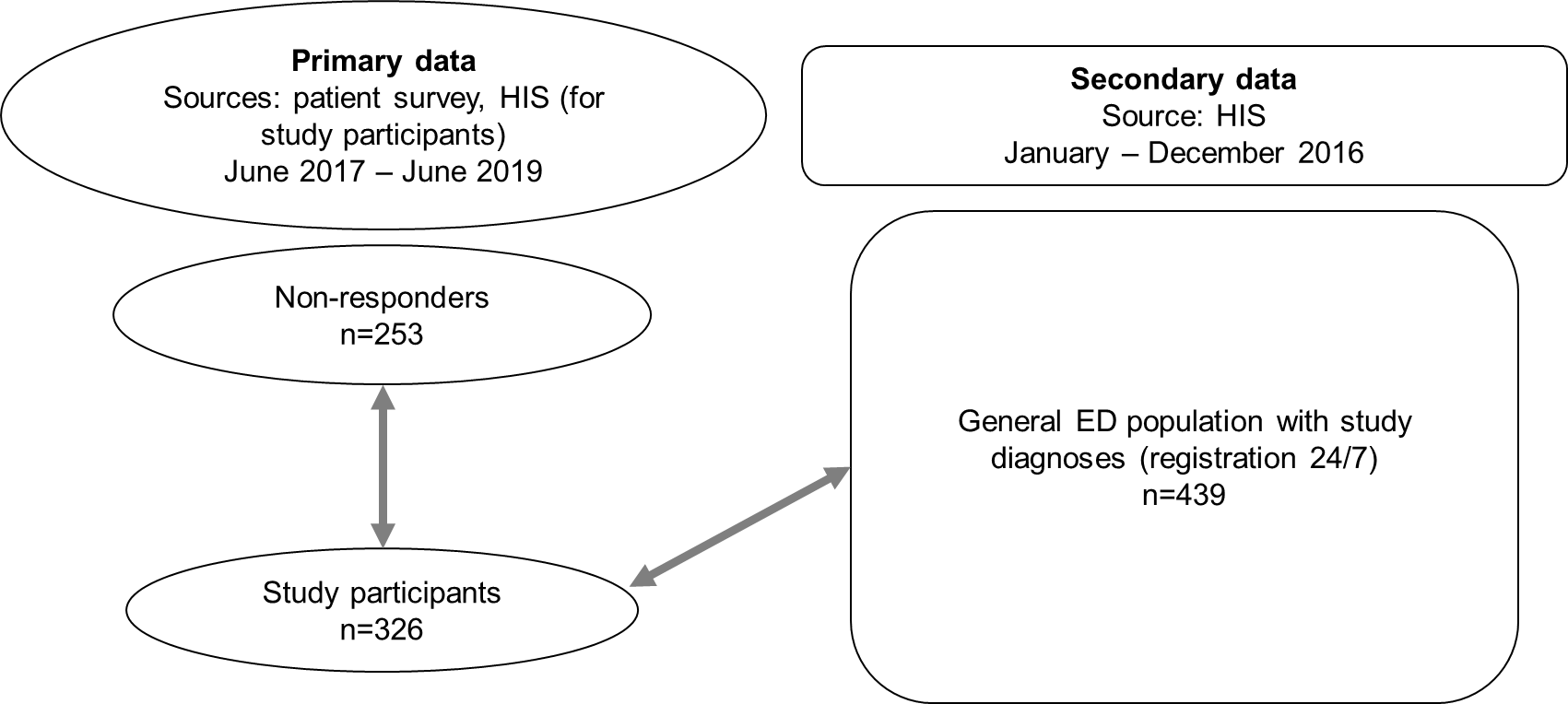


Legend: Ellipses depict the primary data sample and rectangles secondary data samples used for analyses. Arrows between shapes illustrate data samples which were compared numerically in this study. Numbers depict patients with relevant study diagnoses for the research project EMAAGE only. In EMAAGE, patients were recruited on wards following ED treatment without time restrictions. Abbreviations: ED: emergency department, HIS: hospital information system.
