# Supplementary material for: Primary and secondary data in emergency medicine health services research – a comparative analysis in a regional research network on multimorbid patients
Source: BMC Med Res Methodol. 2023 Feb 4;23:34. doi: 10.1186/s12874-023-01855-2 (PMC9898937; doi:10.1186/s12874-023-01855-2)
Supplement: Supplementary file 5 — Additional file 5: Figure 5. Graphical distribution of transportation to ED in primary and secondary data samples in EMAAGE, EMACROSS, and EMASPOT. [file 12874_2023_1855_MOESM5_ESM.docx]

Additional Figure 5: Graphical distribution of transportation to ED in primary and secondary data samples in EMAAGE, EMACROSS, and EMASPOT
