# Supplementary material for: Primary and secondary data in emergency medicine health services research – a comparative analysis in a regional research network on multimorbid patients
Source: BMC Med Res Methodol. 2023 Feb 4;23:34. doi: 10.1186/s12874-023-01855-2 (PMC9898937; doi:10.1186/s12874-023-01855-2)
Supplement: Supplementary file 7 — Additional file 7: Table 1. Description of non-responders and study participants regarding gender and age from screening logs. [file 12874_2023_1855_MOESM7_ESM.docx]

Additional Table 1: Description of non-responders and study participants regarding gender and age from screening logs

| Sub-study | Variable | Non-responders  n (%) / mean (SD) | Study participants  n (%) / mean (SD) |
| --- | --- | --- | --- |
| EMAAge  (n=579) | Male | 107 (42.3) | 107 (32.8) |
|  | Female | 146 (57.7) | 219 (67.2) |
|  | Age in years | 77.4 (13.4) | 75.8 (12.1) |
| EMACROSS  (n=1032) | Male | 274 (48.9) | 251 (53.2) |
|  | Female | 286 (51.1) | 221 (46.8) |
|  | Age in years | 56.0 (19.9) | 53.6 (19.1) |
| EMASPOT  (n=1418) | Male | 440 (56.8) | 376 (58.4) |
|  | Female | 334 (43.2) | 268 (41.6) |
|  | Age in years | 70.1 (11.1) | 68.4 (10.8) |

Note: SD standard deviation.
