# Supplementary material for: Primary and secondary data in emergency medicine health services research – a comparative analysis in a regional research network on multimorbid patients
Source: BMC Med Res Methodol. 2023 Feb 4;23:34. doi: 10.1186/s12874-023-01855-2 (PMC9898937; doi:10.1186/s12874-023-01855-2)
Supplement: Supplementary file 8 — Additional file 8: Table 2. Inclusion criteria for the three EMANET sub-studies EMAAGE, EMACROSS, and EMASPOT. [file 12874_2023_1855_MOESM8_ESM.docx]

Additional Table 2: Inclusion criteria for the three EMANet sub-studies EMAAge, EMACROSS, and EMASPOT

| Inclusion criteria EMAAge:   - Age ≥ 18 years   AND   - Presence of at least one of the following suspected ICD-10 diagnoses in the ED:   - S72.0 Fracture of neck of femur   - S72.1 Pertrochanteric fracture   - S72.2 Subtrochanteric fracture |
| --- |
| Inclusion criteria EMACROSS:   - Age ≥ 18 years   AND   - Presence of at least one of the following suspected ICD-10 diagnoses in the ED:   - J09 Influenza due to identified zoonotic or pandemic influenza virus   - J10 Influenza due to identified seasonal influenza virus   - J11 Influenza, virus not identified   - J12 Viral pneumonia, not elsewhere classified   - J13 Pneumonia due to Streptococcus pneumonia   - J14 Pneumonia due to Haemophilus influenza   - J15 Bacterial pneumonia, not elsewhere classified   - J16 Pneumonia due to other infectious organisms, not elsewhere classified   - J17 Pneumonia in diseases classified elsewhere   - J18 Pneumonia, organism unspecified   - J20 Acute bronchitis   - J21 Acute bronchiolitis   - J22 Unspecified acute lower respiratory infection   - J40 Bronchitis, not specified as acute or chronic   - J41 Simple and mucopurulent chronic bronchitis   - J42 Unspecified chronic bronchitis   - J43 Emphysema   - J44 Other chronic obstructive pulmonary disease   - J45 Asthma   - J46 Status asthmaticus   - J47 Bronchiectasis   OR   - Presence of at least one of the following main symptoms at ED presentation:   - Cough   - Shortness of breath / Dyspnea   - Expectoration   - Fever   - Common cold   - Sore throat   - Thoracic pain (in conjunction with respiratory complaints)   - Earache   - Fatigue   - Melalgia (in conjunction with respiratory complaints) |
| Inclusion criteria EMASPOT   - Age ≥ 50 years   AND   - Presence of at least one of the following suspected ICD-10 diagnoses in the ED:   - I10 Essential (primary) hypertension   - I11.0 Hypertensive heart disease with (congestive) heart failure / I11.9 Hypertensive heart disease without (congestive) heart failure   - I20 Angina pectoris   - Excluded diagnosis of I21 Acute myocardial infarction   - I24.0 Coronary thrombosis not resulting in myocardial infarction / I24.8 Other forms of acute ischaemic heart disease / I24.9 Acute ischaemic heart disease, unspecified   - I47.1 Supraventricular tachycardia / I47.9 Paroxysmal tachycardia, unspecified   - I48.0 Paroxysmal atrial fibrillation / I48.2 Chronic atrial fibrillation / I48.9 Atrial fibrillation and atrial flutter, unspecified   - I49.5 Sick sinus syndrome / I49.8 Other specified cardiac arrhythmias / I49.9 Cardiac arrhythmia, unspecified   - I50 Heart failure   - J81 Pulmonary oedema   - R00.0 Tachycardia, unspecified / R00.1 Bradycardia, unspecified / R00.2 Palpitations / R00.8 Other and unspecified abnormalities of heart beat   - R07.3 Other chest pain / R07.4 Chest pain, unspecified   OR   - Presence of at least one of the following main symptoms at ED presentation:   - Chest pain   - Chest tightness   - Shortness of breath / Dyspnea   - Malaise   - Nausea   - Dizziness   - Weakness   - Fatigue   - Asthenia   - Problems with blood pressure   - Tachycardia   - Extrasystole   - Cardiac flutter   - Arrhythmia   - Swollen legs   - Weight gain   - Increase in abdominal girth |

Note: ICD-10 International Statistical Classification of Diseases and Related Health Problems 10th Revision.
