# Supplementary material for: Primary and secondary data in emergency medicine health services research – a comparative analysis in a regional research network on multimorbid patients
Source: BMC Med Res Methodol. 2023 Feb 4;23:34. doi: 10.1186/s12874-023-01855-2 (PMC9898937; doi:10.1186/s12874-023-01855-2)
Supplement: Supplementary file 9 — Additional file 9: Table 3. Description of missing data in primary and secondary data samples in MTS category, transportation to ED, discharge type, and case type. [file 12874_2023_1855_MOESM9_ESM.docx]

Additional Table 3: Description of missing data in primary and secondary data samples in MTS category, transportation to ED, discharge type, and case type

| EMAAGE | | | | |
| --- | --- | --- | --- | --- |
|  | primary data sample | missing rate (in %) | secondary data sample | missing rate (in %) |
| Total (n) | 326 |  | 439 |  |
|  |  |  |  |  |
| MTS category | 58 | 17.8 | 186 | 42.4 |
| Transportation to ED | 17 | 5.2 | 72 | 16.4 |
| Discharge type | 10 | 3.1 | 14 | 3.2 |
|  |  |  |  |  |
| EMACROSS | | | | |
|  | primary data sample | missing rate (in %) | secondary data sample | missing rate (in %) |
| Total (n) | 472 |  | 3410 |  |
|  |  |  |  |  |
| MTS category | 16 | 3.4 | 1572 | 46.1 |
| Transportation to ED | 10 | 2.1 | 977 | 28.7 |
| Case type |  |  | 178 | 5.2 |
| Discharge type | 27 | 5.7 | 1260 | 37.0 |
|  |  |  |  |  |
| EMASPOT | | | | |
|  | primary data sample | missing rate (in %) | secondary data sample | missing rate (in %) |
| Total (n) | 644 |  | 5480 |  |
|  |  |  |  |  |
| MTS category | 33 | 5.1 | 2281 | 41.6 |
| Transportation to ED | 38 | 5.9 | 1022 | 18.6 |
| Case type |  |  | 33 | 0.6 |
| Discharge type | 32 | 5.0 | 1647 | 30.1 |

Note: ED emergency department; MTS Manchester Triage System.
